# Effects of bleaching agents containing nano-hydroxyapatite on sound and demineralized enamel: an in vitro study

**DOI:** 10.1007/s10266-026-01328-0

**Published:** 2026-01-30

**Authors:** Merve Haberal, Çiğdem Çelik

**Affiliations:** Department of Restorative Dentistry, Faculty of Dentistry, Kirikkale University, Kirikkale, Turkey

**Keywords:** Dental bleaching, Nano-hydroxyapatite, Surface roughness, Surface microhardness, SEM

## Abstract

This in vitro study aimed to evaluate the effects of office and home bleaching agents containing nano-hydroxyapatite (n-HAP) on the surface roughness, microhardness, and micromorphology of sound and demineralized enamel. A total of 120 enamel specimens were obtained from extracted human third molars, half of which underwent a demineralization cycle to simulate initial enamel demineralization. The specimens were divided into five subgroups (*n* = 12) based on the bleaching agent applied: no bleaching (control), 40% hydrogen peroxide (HP), 16% carbamide peroxide (CP), 40% HP + n-HAP, and 6% HP + n-HAP. Surface roughness was measured using non-contact optical profilometry, and microhardness was assessed via the Vickers hardness test. Micromorphological changes were examined by scanning electron microscopy (SEM) at × 1,000 and × 4,000 magnifications. Statistical analyses were performed using the Shapiro–Wilk, Mann–Whitney *U*, Kruskal–Wallis H, and Bonferroni post hoc tests (*p* < 0.05). In the demineralized groups, surface roughness significantly increased while microhardness decreased (*p* < 0.05). The highest surface roughness was observed in the demineralized group treated with 16% CP, whereas microhardness significantly increased in the group treated with 6% HP + n-HAP. SEM findings revealed pronounced structural degradation in groups treated with high-concentration HP, while n-HAP–containing groups exhibited mineral deposition and preservation of the prismatic enamel structure. Although no remineralization protocol was used before bleaching, the findings indicate that low-concentration HP agents containing n-HAP may protect enamel with initial demineralization. These results highlight the potential clinical benefit of such formulations in reducing bleaching-related enamel damage.

## Background

When patients present to dental clinics for a whiter, esthetically pleasing smile, clinicians today refer them to tooth-bleaching treatments as an alternative to invasive restorations [1, 2]. Hydrogen peroxide (HP) is used as an active agent in such treatment because it releases reactive oxygen molecules, and sodium perborate or carbamide peroxide (CP) may also be chemically reacted to make HP [3]. Due to the demineralization that occurs during tooth bleaching, microscopic surface defects and subsurface pores form in the enamel [4]. The mechanism of action of HP molecules can disrupt tooth morphology and structure, and this change in surface morphology decreases the microhardness value and increases roughness [5, 6].

Demineralization is the removal of mineral ions from hydroxyapatite crystals in the structure of hard tissues, such as enamel, dentin, cement, and bone. In contrast, the recovery of mineral ions is termed remineralization. Partially demineralized hydroxyapatite crystals in dental tissue can return to their original size when exposed to environmental conditions that support remineralization. Saliva, fluoride applications, dietary control, probiotics, bioactive glass, and a number of compounds containing nano-hydroxyapatite (n-HAP) are defined as agents that promote remineralization [7].

N-HAP is a synthetic form of hydroxyapatite that closely resembles dental enamel and has a strong affinity for demineralized surfaces. It adheres to areas damaged by acid and clusters on the tooth surface, forming a coating that fully integrates with both prismatic and interprismatic enamel [8]. N-HAP particles are used in various treatments, such as remineralization of hypomineralized enamel defects and sensitivity reduction. The size of the n-HAP particles allows the material to pass through microcracks to reach the dentinal tubules. Therefore, the deposition of n-HAP molecules causes the cracks to narrow [9]. The use of remineralization agents containing n-HAP has also been shown to reduce hypersensitivity during tooth-bleaching treatment [10].

Recently, bleaching agents containing n-HAP have been introduced to improve the safety and biocompatibility of conventional peroxide-based bleaching systems [11, 12]. Unlike traditional bleaching formulations that rely solely on the oxidative effect of hydrogen peroxide, agents containing nano-hydroxyapatite combine whitening efficacy with a remineralizing potential during the bleaching process [13]. Due to its chemical similarity to natural enamel, n-HAP can penetrate enamel porosities, promote the deposition of calcium and phosphate ions, and contribute to the partial restoration of mineral balance [14]. This bioactive interaction may help preserve enamel surface integrity, reduce post-bleaching sensitivity through the occlusion of interprismatic spaces, and improve surface characteristics [11, 13]. Therefore, bleaching agents containing n-HAP represent a promising approach for achieving effective tooth bleaching while minimizing adverse effects on enamel structure [12, 14].

The evaluation of surface roughness and microhardness provides essential insight into the structural and mechanical integrity of enamel following bleaching procedures [15–18]. Microhardness is closely associated with the mineral content and mechanical resistance of enamel, both of which may be adversely affected by peroxide-based bleaching agents [16, 19]. Surface roughness reflects alterations in enamel topography that can facilitate plaque retention, discoloration, and increased susceptibility to wear or acid erosion [20, 21]. Previous studies have shown that bleaching-induced demineralization may result in decreased hardness and increased surface irregularities, leading to compromised enamel smoothness and optical properties [22–24]. Therefore, the combined assessment of surface roughness and microhardness enables a comprehensive evaluation of the effects of bleaching and remineralizing agents on enamel quality and surface morphology [15].

Dental hard tissues are inherently susceptible to demineralization throughout life. Repeated short-term exposure to acidic challenges may induce early enamel demineralization that often remains clinically undetectable; however, such subclinical mineral loss may be present in patients seeking tooth bleaching and may be further exacerbated by bleaching procedures [25]. From a clinical perspective, the diagnosis of initial demineralization is challenging, and only a limited number of studies have evaluated the effects of tooth-bleaching agents on demineralized enamel surfaces [18, 26, 27]. Moreover, the influence of bleaching agents containing bioactive components, such as n-HAP, which are intended to exert protective or remineralizing effects on dental hard tissues, has not yet been fully elucidated [7, 24, 28]. Therefore, investigating the surface changes induced by different bleaching agents on both sound and demineralized enamel surfaces is of considerable clinical and scientific relevance [29].

This study aimed to evaluate the effects of office and home bleaching agents containing 40% HP, 40% HP + n-HAP, 16% CP, and 6% HP + n-HAP on the surface microhardness and surface roughness of sound and demineralized enamel as well as to examine the morphological changes using scanning electron microscopy (SEM). The null hypothesis was that there was no difference between the microhardness and surface roughness values of home and office bleaching agents with and without n-HAP on sound and demineralized enamel.

## Methodology

**Fig. 1:**
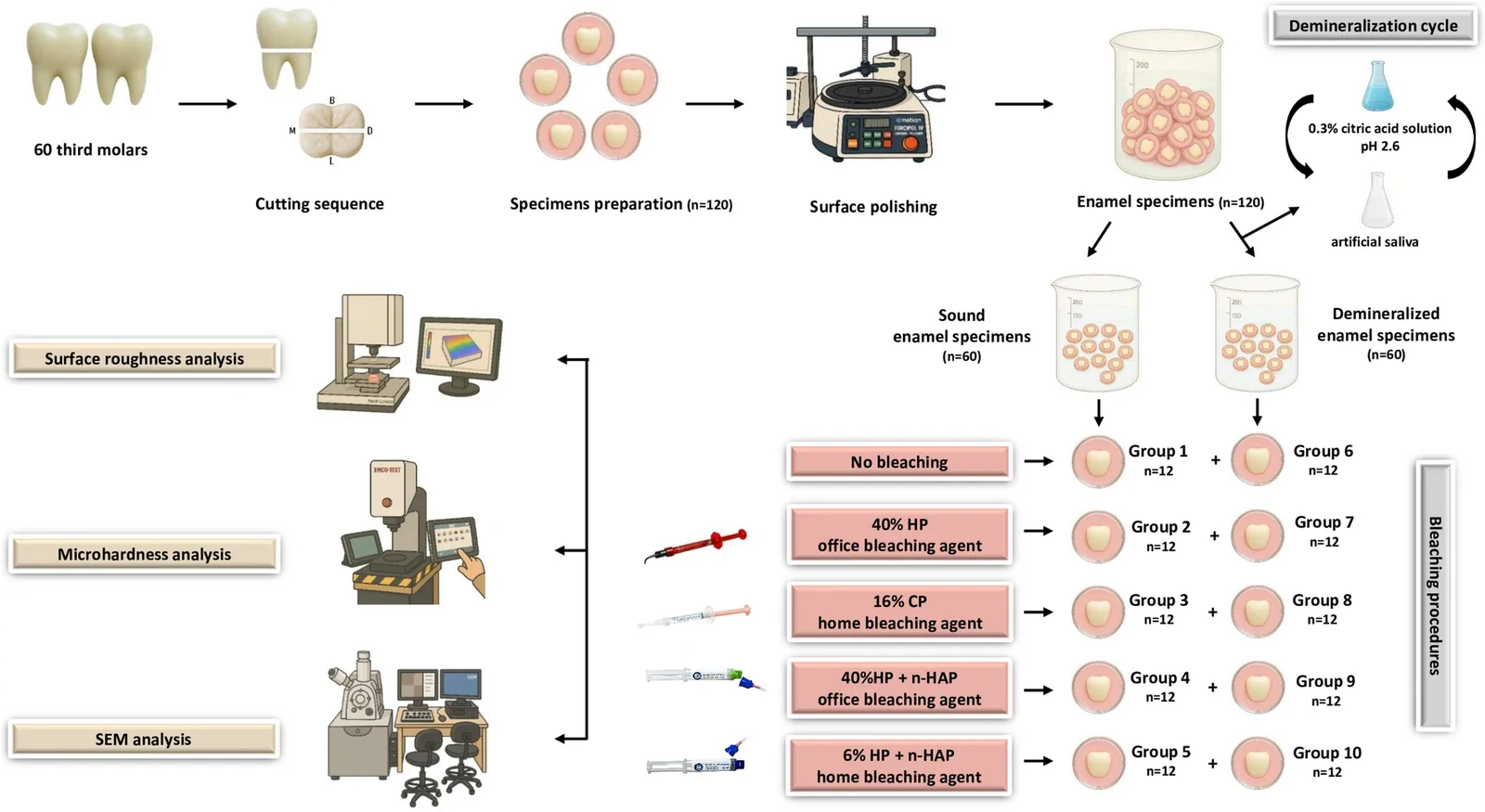
Schematic of study design (B: buccal, D: distal, L: lingual, M: mesial, SEM: scanning electron microscopy)

This study was conducted at Kirikkale University, Faculty of Dentistry, Department of Restorative Dentistry. It was approved by the Kirikkale University Non-Interventional Research Ethics Committee on 21 Dec. 2022 under meeting number 2022/15, with decision number 2022.12.14. The Kirikkale University Scientific Research Projects Coordination Unit provided funding for the study under project code 2023/020. In the preliminary statistical evaluation at the study’s planning stage, the power analysis was calculated using G*Power v.3.1 software (Heinrich Heine, University of Dusseldorf, Dusseldorf, Germany). It was determined that a minimum of 12 specimens should be taken for each group at a medium effect size of 85% power and α (type I) and β (type II) error levels. The schematic of the study design appears in Fig. 1.

### Specimen preparation

This study used 60 sound human third molars that had recently been extracted for periodontal or orthodontic purposes. Informed consent was obtained from all patients prior to the use of their extracted teeth in the study. Teeth with caries, fractures/cracks, discoloration, hypocalcified/hypomineralized areas, or defects were excluded. The hard and soft tissue remnants on the chosen teeth were eliminated with a sharp excavator. Until the experimental phase, the teeth were maintained in a 0.1% thymol solution at + 4 °C in a refrigerator. The solution was changed weekly. The crowns and roots of the teeth were separated at the cemento-enamel junction with a slow-speed diamond saw (IsoMet, Buehler Ltd., USA) mounted on an electric precision cutter (Micracut 151, Metkon, Turkey). Using the same technique, the crowns were bisected mesiodistally to create 120 enamel specimens from the middle third of the buccal and lingual surfaces of the teeth. The specimens were embedded in plexiglass molds using polymethyl methacrylate-based cold acryl (Integra Self-Cure Acrylic-Liquid, BGD, Turkey) with the enamel surfaces localized on top. The enamel surfaces were polished with 600-, 1200-, 2400-, and 4000-grit aluminum oxide abrasives (Hermes/Schleifmittel GmbH & Co., Hamburg, Germany) with a polishing device (Forcipol 1 V Grinder Polisher, Metkon, Turkey) for 40 s under water cooling. The specimens were then washed with distilled water for 1 min to obtain smooth, shiny, residue-free enamel surfaces. The specimens were kept in artificial saliva at 37 °C in an incubator.

### Preparation of demineralization and artificial saliva solutions

In this study, 0.3% citric acid solution with pH = 2.6 was used for demineralization [30, 31]. The artificial saliva components were prepared according to the formula for 1000 ml deionized water (g/L): 0.213 g CaCl_2_·2H_2_O, 0.738 g KH_2_PO_4_, 1.114 g KCl, 0.381 g NaCl, and 12 g Tris buffer [32].

### Demineralization cycle

Half the study specimens underwent a demineralization cycle. The demineralization protocol used in this study was designed to simulate the early-stage alterations of enamel erosion. For this purpose, short and repeated applications of 0.3% citric acid (pH = 2.6) were employed, which induce superficial mineral loss characterized by partial dissolution of the aprismatic enamel layer without producing subsurface caries-like lesions. The specimens were submerged for 5 min in a 0.3% citric acid solution for this procedure, after which they were kept in an artificial saliva solution for 60 min. This procedure was carried out 4 times a day for 5 days [30, 31].

### Bleaching procedures

Table 1 shows the composition, manufacturer, and lot numbers of the products used in the present study. The sound and demineralized enamel specimens were each divided into five subgroups (*n* = 12) according to bleaching agent. All procedures in the study were conducted by a single investigator to guarantee consistency, and they complied with the manufacturer’s instructions.

**Table 1:** Composition, manufacturer and, LOT number of the products used

Product	Manufacturer	LOT number	Composition
Opalescence BOOST PF 40%; office bleaching agent	Ultradent Products, South Jordan, UT, USA	BP979	40% hydrogen peroxide, potassium nitrate, fluoride, water
Opalescence PF 16%; home bleaching agent	Ultradent Products, South Jordan, UT, USA	BPBR3 BPT18	16% carbamide peroxide, potassium nitrate, fluoride, water
BioWhiten ProOffice PowerHP 40%; office bleaching agent	Biodent Ltd., Istanbul, Turkey	K40H2301	40% hydrogen peroxide, nano-hydroxyapatite, sodium bicarbonate, sodium hydroxide, water
BioWhiten ProHome 6%; home bleaching agent	Biodent Ltd., Istanbul, Turkey	K06HJ2211	6% hydrogen peroxide, nano-hydroxyapatite, sodium bicarbonate, sodium hydroxide, water

The specimens in Group (G) 1, 2, 3, 4, and 5 were not subjected to any process, whereas the demineralization cycle was applied to the specimen surfaces of Group (G) 6, 7, 8, 9, and 10. The bleaching procedures applied in the test groups are described below.

#### G1 (positive control)/G6 (negative control)

No bleaching procedure was applied.

#### G2/G7

Forty percent HP office bleaching agent (Opalescence Boost, Ultradent, South Jordan, UT, USA) was applied to the enamel surfaces at a thickness of 1 mm in accordance with the manufacturer’s instructions. To ensure a uniform application in all groups, the thickness of the bleaching gel was verified using a graduated measuring probe at four peripheral points and the center of each specimen, confirming a mean thickness of 1 mm before treatment. After 20 min on the enamel surface, it was aspirated from the surface, and this process was repeated twice. The bleaching agent was mixed with an applicator at 5-min intervals during the procedure. The specimen surfaces were completely cleaned using an air–water spray at the conclusion of the procedure.

#### G3/G8

The enamel surfaces were treated with a 1 mm layer of home bleaching agent containing 16% CP (Opalescence PF 16%, Ultradent, South Jordan, UT, USA). The agent was washed off with air–water spray after 4 h on the enamel surfaces. The specimens were kept in artificial saliva at 37 °C during the bleaching procedure. The procedure was repeated for 7 days in total.

#### G4/G9

The office bleaching agent containing 40% HP + n-HAP (BioWhiten ProOffice PowerHP 40%, Biodent Ltd., Istanbul, Turkey) was applied at 1 mm thickness to the enamel surface. The agent was mixed with the help of an applicator at intervals of 5 min. The bleaching agent was kept on the enamel surface for 15 min in accordance with the manufacturer’s instructions and removed by aspirating. After this process was repeated three times consecutively, the specimens were washed thoroughly with air–water spray.

#### G5/G10

The home bleaching agent containing 6% HP + n-HAP (BioWhiten ProHomeWhitening 6%, Biodent Ltd., Istanbul, Turkey) was applied to the enamel surfaces at a thickness of 1 mm and left for 60 min in accordance with the manufacturer’s instructions. Afterward, the enamel surface was washed with an air–water spray, and the specimens were placed in artificial saliva solution at 37 °C. This process was repeated for a total of 14 days.

All the test groups were stored in distilled water at 37 °C until the surface analyses began.

### Surface roughness analysis

After the bleaching procedures, the specimens were analyzed for surface roughness using a non-contact optical profilometer (Profilm 3D, Filmetrics, San Diego, CA, USA). The samples were air-dried, and a 2 × 2 mm area was selected at the center of each enamel surface. Surface profiles were scanned using white light interferometry at a sensitivity of 0.05 μm. The raw data were processed using ProfilmOnline software (KLA Corp., Milpitas, CA, USA) to determine the surface roughness parameters. Both the roughness average (Ra) and the areal surface roughness (Sa) values were measured. Ra is a two-dimensional (2D) line-based roughness parameter representing the arithmetic mean of the absolute deviations of the surface profile from the mean line and was calculated as the average of three linear scans obtained across the measured area. In contrast, Sa is a three-dimensional (3D) areal roughness parameter, defined as the arithmetic mean height of the surface over the entire measured area, and was analyzed to provide a comprehensive evaluation of enamel surface topography.

### Microhardness analysis

After the specimens’ surface roughness examinations, surface microhardness measurements were performed with a DuraScan 20 G5 (Emco Test, Kuchl-Salzburg, Austria). The specimens were placed on the table of the device after drying with air spray. Using the Vickers measurement method, the diagonals of the quadrilateral scar left by the diamond square pyramid tip with a peak angle of 136° (which was pressed against the material with a load of 50 g) were measured. The Vickers microhardness values (HVs) of the specimens were obtained by inserting the diagonal length into the formula HV = (kgf/mm^2^) = constant (test force F/surface of indentation) = 0.102 (2F sin 136°/2)/*d*^2^ ≈ 0.1891(*F*⁄*d*^2^). In this formula, *d* is the average diagonal length of the track as determined by the formula *d* = (*d*1 + *d*2)⁄2. The HVs obtained from five randomly selected points on each specimen surface were averaged.

### Scanning electron microscopy (SEM) analysis

Three randomly selected specimens from each group were examined using a Hitachi SU-1500 scanning electron microscope (SEM) (Hitachi Co., Tokyo, Japan) to identify morphological changes, such as microvoids, cracks, melting, or thermal damage zones on the surface. All specimens were placed in an oven (Nüve Incubator FN 055, Ankara, Turkey) at 37 °C for 24 h to dry. Before the SEM examination, the specimens were fixed on the discs with double-sided carbon adhesive. In the SEM evaluations, the specimen surfaces were coated with 10 nm–thick gold palladium in a surface coating device (Denton Vacuum Desk V, Moorestown, NJ, USA). The coating process was carried out in 30 s under a vacuum pressure of 0.07 torr using a current of 52 mA. The SEM examinations were performed at magnifications of × 1,000 and × 4,000.

### Statistical analysis

The data were analyzed using IBM SPSS 22 (SPSS Statistics, Chicago, USA). Compliance with a normal distribution was assessed by the Shapiro–Wilk test. Because the data were not normally distributed, the Kruskal–Wallis H test was used for comparisons involving three or more groups and the Mann–Whitney *U*-test for comparisons involving two groups. The Bonferroni post hoc test was used for multiple comparisons. The significance level was determined as 0.05.

## Results

### Surface roughness evaluation

**Fig. 2:**
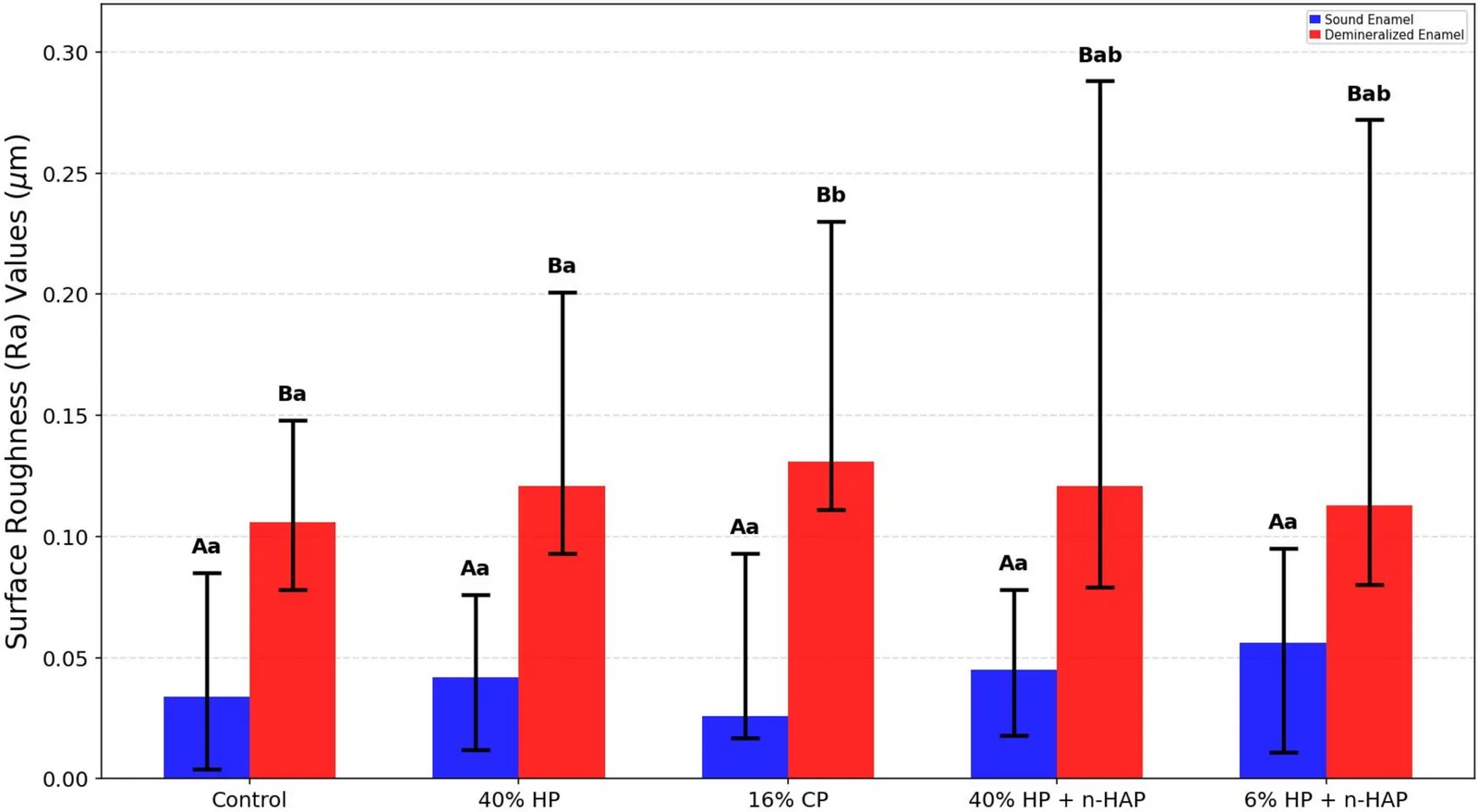
Median (minimum–maximum) surface roughness values of sound and demineralized enamel after the application of different bleaching agents, expressed as Ra (roughness average, µm). *Values sharing the same uppercase letter indicate no statistically significant difference between different enamel surfaces within the same applied bleaching agent group, whereas values sharing the same lowercase letter indicate no statistically significant difference among different applied bleaching agents within the same enamel surface (*p* > 0.05)

**Fig. 3:**
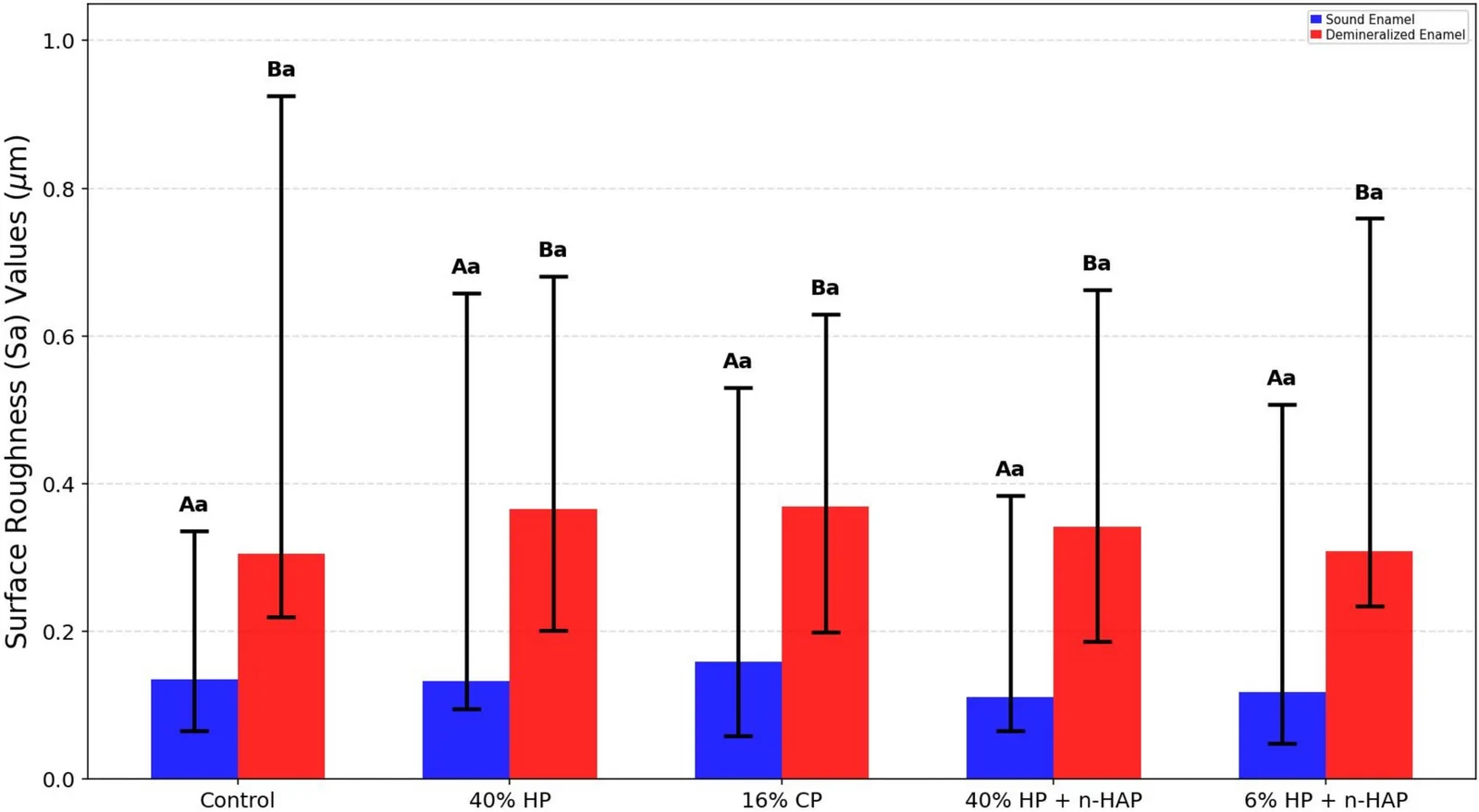
Median (minimum–maximum) surface roughness values of sound and demineralized enamel following different bleaching protocols, expressed as Sa (areal surface roughness, µm). *Values sharing the same uppercase letter indicate no statistically significant difference between different enamel surfaces within the same applied bleaching agent group, whereas values sharing the same lowercase letter indicate no statistically significant difference among different applied bleaching agents within the same enamel surface (*p* > 0.05)

The surface roughness values expressed as Ra for sound and demineralized enamel after the application of different bleaching agents are illustrated in the graph shown in Fig. 2. A statistically significant difference was found between the surface roughness values of the sound and demineralized specimens, indicating that the demineralization cycle increased the surface roughness values in all groups (*p* < 0.05). There were no statistically significant differences between the surface roughness values of the sound enamel groups (*p* > 0.05), but a significant difference was found in the surface roughness values of the demineralized groups (*p* < 0.05). Pairwise comparisons revealed that the group treated with a home bleaching agent containing 16% CP had a roughness value significantly higher than that of the negative control group and the group treated with an office bleaching agent containing 40% HP (*p* = 0.018, *p* = 0.032). The areal surface roughness values expressed as Sa for sound and demineralized enamel after the application of different bleaching agents are illustrated in the graph shown in Fig. 3. Similar to the Ra findings, it was observed that the demineralization cycle significantly increased the areal surface roughness of enamel, regardless of the applied bleaching protocol (*p* < 0.05). In contrast, no statistically significant differences in Sa values were detected among the sound and demineralized enamel groups treated with different bleaching agents (*p* > 0.05). Although the difference was not statistically significant, similar to the Ra findings, the lowest Sa value among the demineralized enamel groups was observed in the control group, whereas the highest Sa value was recorded in the group with 16% CP applied.

**Fig. 4:**
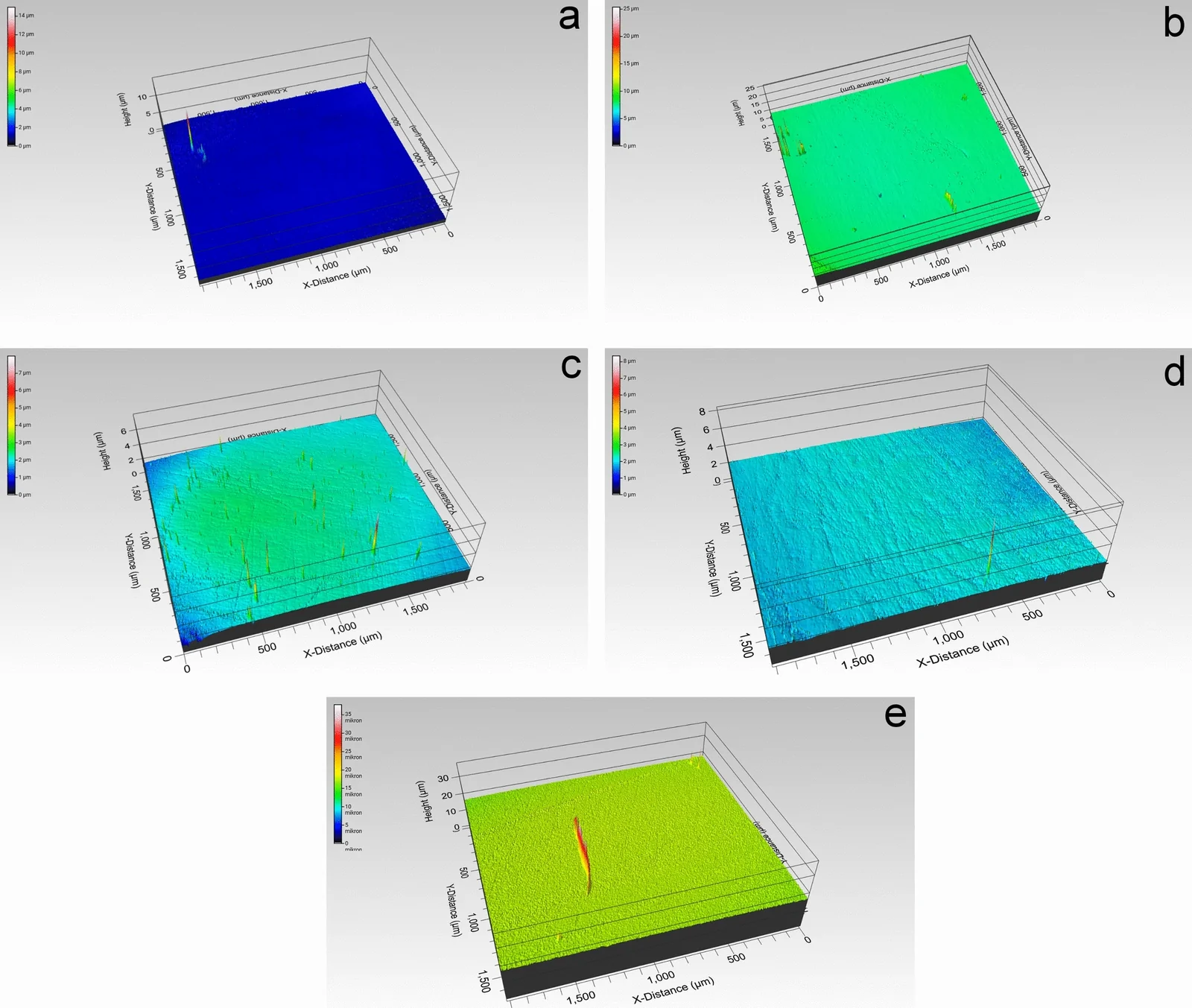
Three-dimensional (3D) optical profilometer images of randomly selected specimens from the sound enamel groups (a = G1, b = G2, c = G3, d = G4, e = G5)

**Fig. 5:**
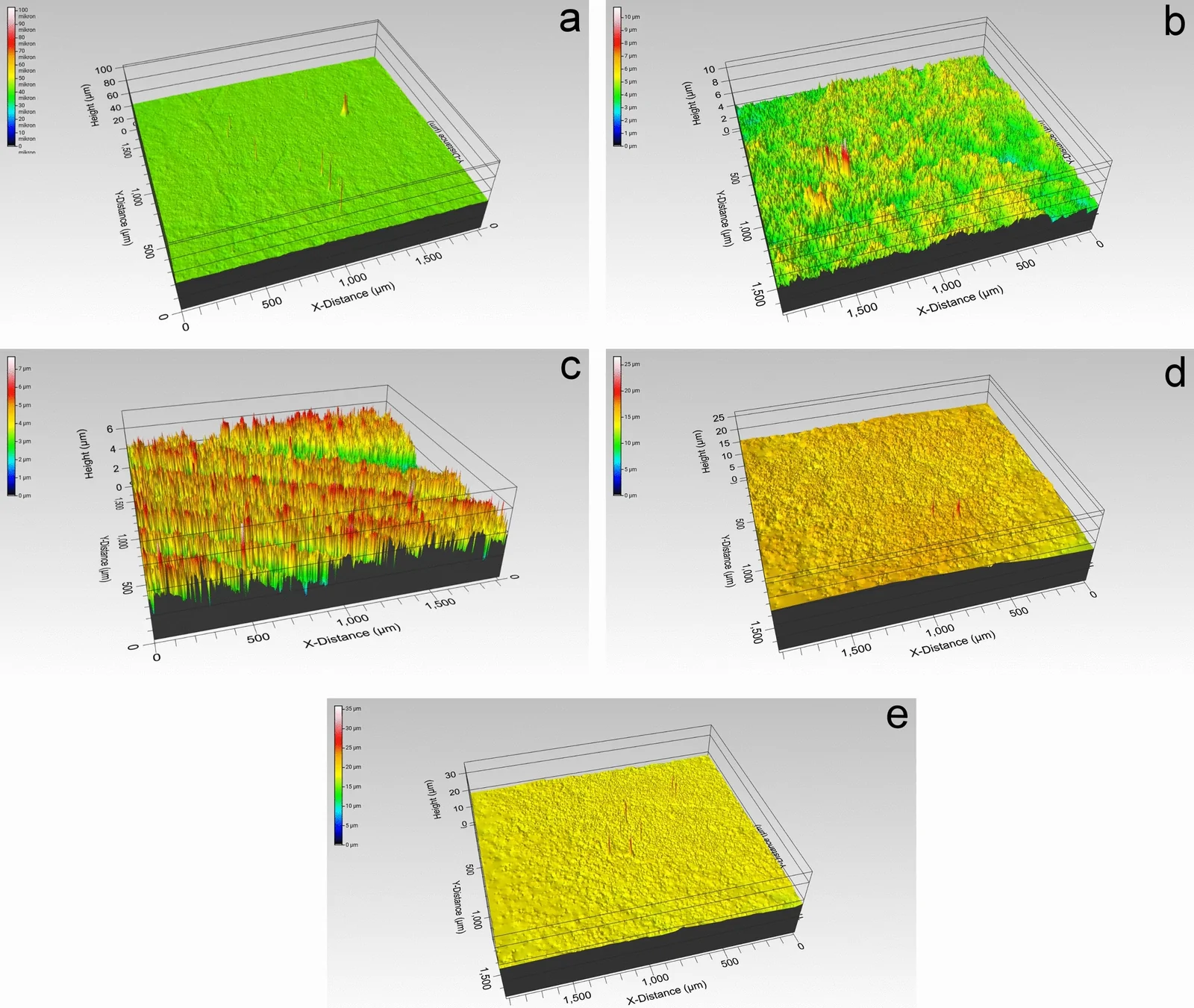
Three-dimensional (3D) optical profilometer images of randomly selected specimens from the demineralized enamel groups (a = G6, b = G7, c = G8, d = G9, e = G10)

When the 3D surface structure was visually evaluated, the surface structure showed similar roughness characteristics in the images of the sound enamel groups (Fig. 4). A considerably rougher image was observed in the groups with demineralization cycles compared to the groups without demineralization cycles (Fig. 5). The application of a home bleaching agent containing 16% CP to demineralized enamel produced the highest surface roughness among all groups, as evidenced by profilometric data and surface topography imaging (Fig. 5c).

### Microhardness evaluation

**Fig. 6:**
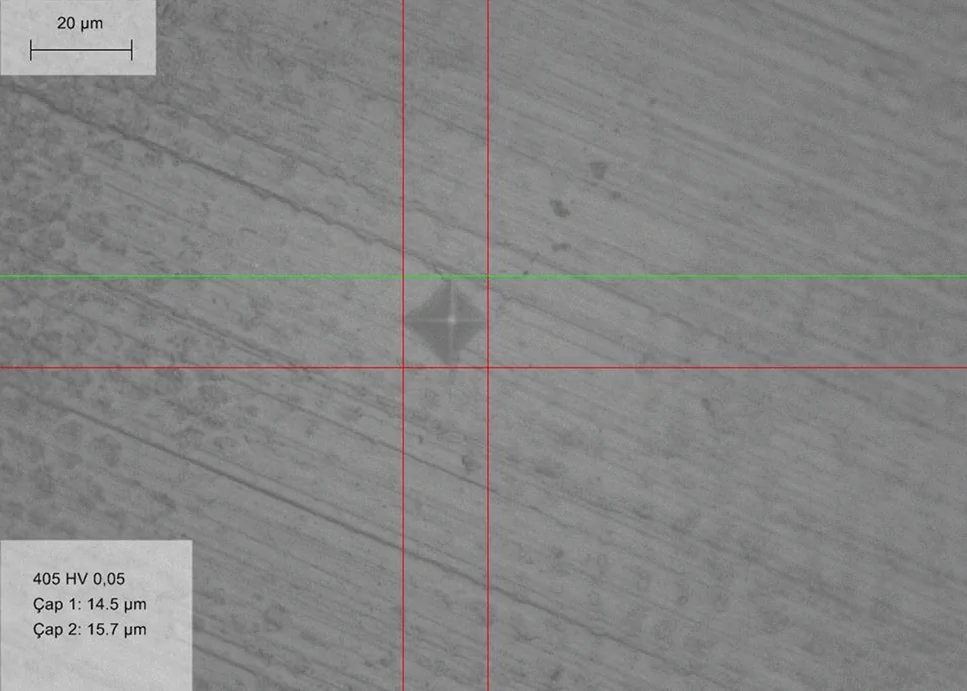
The micrograph corresponding to the Vickers microhardness measurement at × 50 magnification

**Fig. 7:**
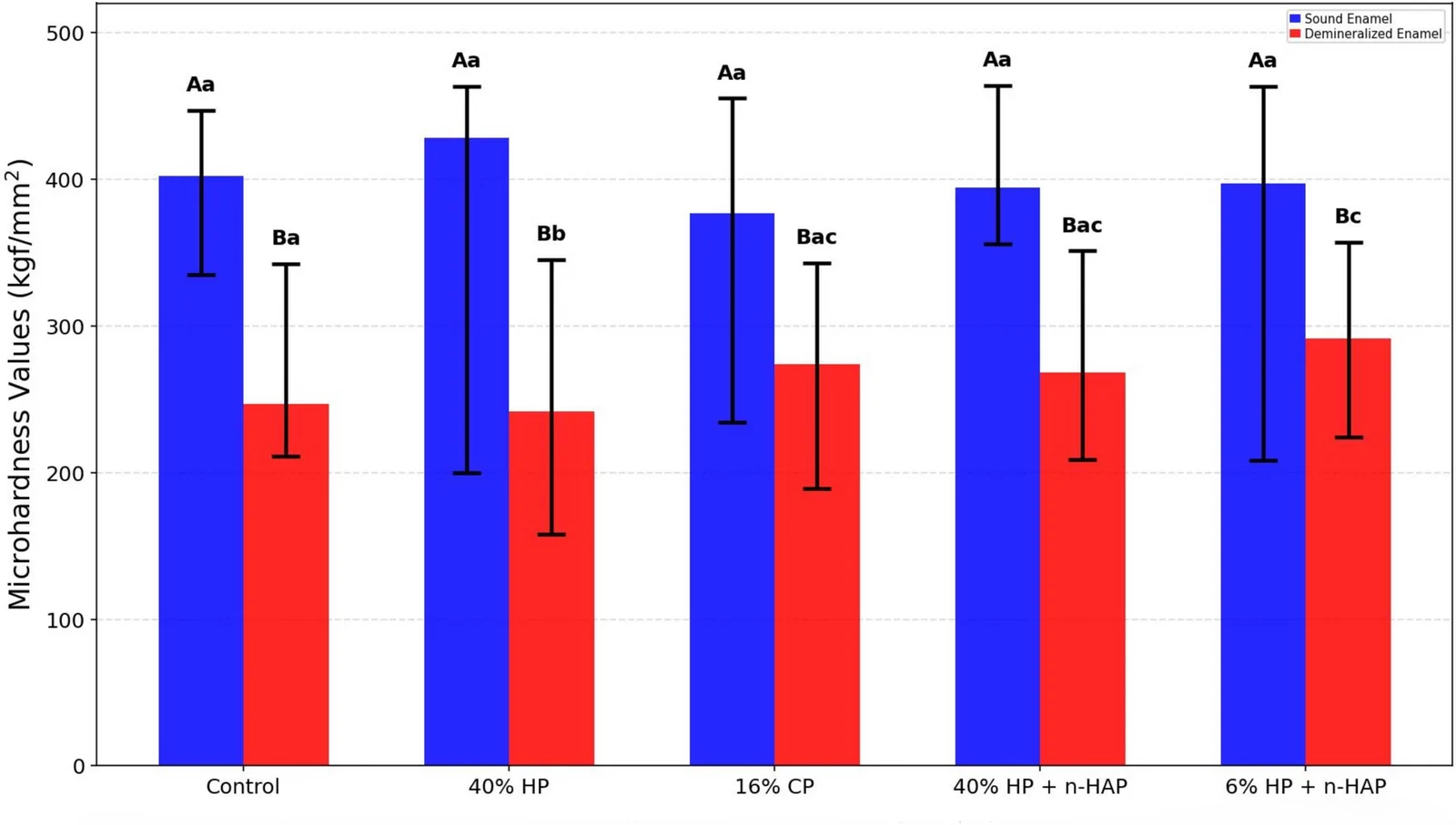
Median (minimum–maximum) microhardness values (kgf/mm.^2^) of sound and demineralized enamel following different bleaching protocols. *Values sharing the same uppercase letter indicate no statistically significant difference between different enamel surfaces within the same applied bleaching agent group, whereas values sharing the same lowercase letter indicate no statistically significant difference among different applied bleaching agents within the same enamel surface (*p* > 0.05)

Figure 6 shows the Vickers microhardness measurement at × 50 magnification. The microhardness values of sound and demineralized enamel following different bleaching protocols are illustrated in the graph shown in Fig. 7. The data reveal that the microhardness values measured in all the demineralization groups were significantly lower than those in the sound enamel groups (*p* < 0.05), but there were no statistically significant differences among the sound enamel groups’ microhardness measurements (*p* > 0.05). In the demineralization groups, the microhardness value of the group treated with an office bleaching agent containing 40% HP was significantly lower than that of the other groups (G7/G6: *p* = 0.021; G7/G8: *p* = 0.031; G7/G9: *p* = 0.038; G7/G10: *p* = 0.002). Furthermore, a statistically significant difference was found between the microhardness values of the groups treated with home bleaching agent containing 6% HP + n-HAP and the corresponding values of the negative control group (*p* = 0.004).

### SEM evaluation

**Fig. 8:**
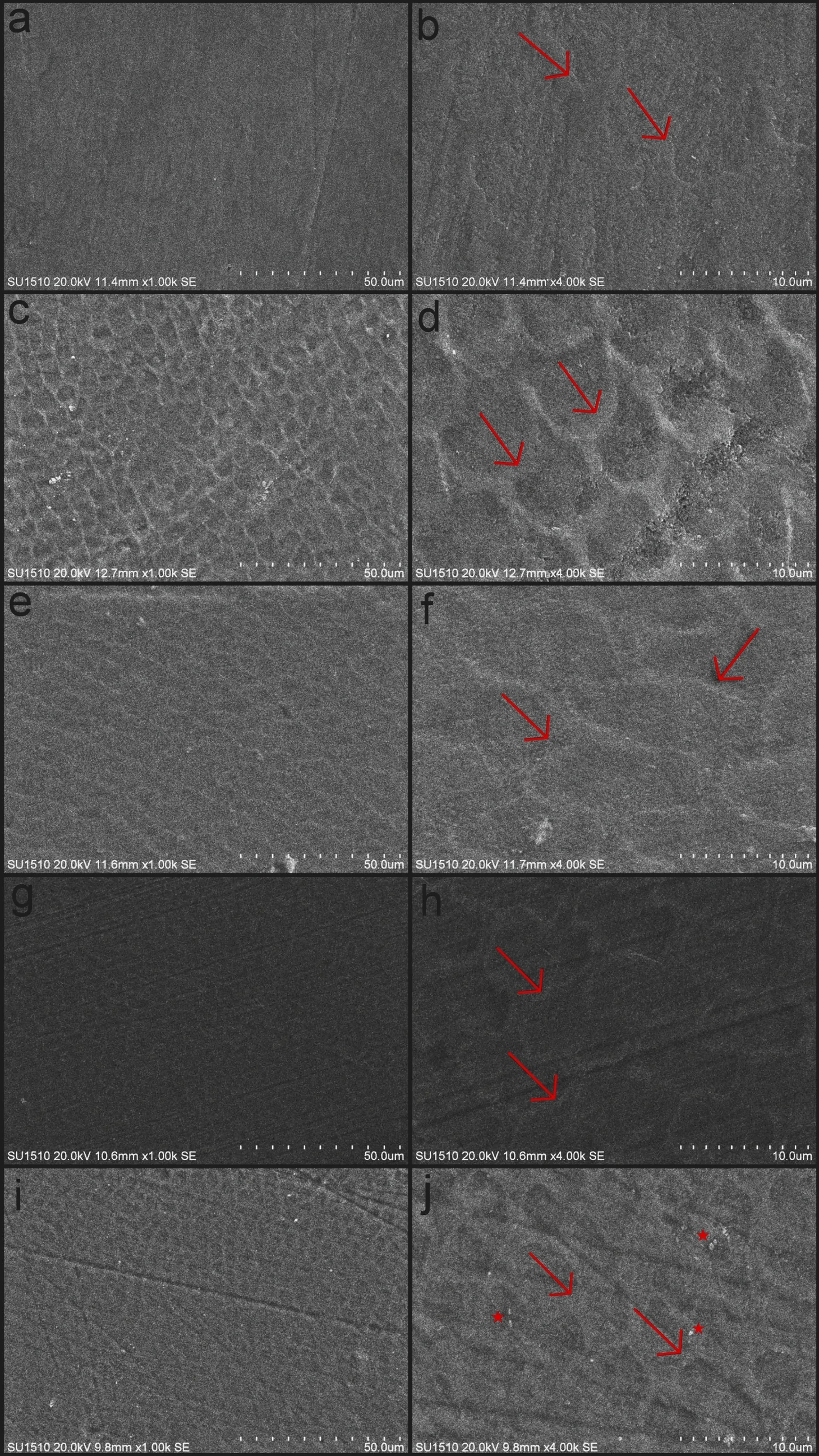
SEM micrographs of randomly selected specimens from groups G1 to G5 at × 1,000 and × 4,000 magnifications (left to right). G1 (**a**–**b**): red arrow: aprismatic layer on sound enamel surface; G2 (**c**–**d**): red arrow: rod and interrods seen after bleaching; G3 (**e**–**f**): red arrow: rod and interrods seen after bleaching; G4 (**g**–**h**): red arrow: rod and interrods seen after bleaching; G5 (**i**–**j**): red arrow: rod and interrods seen after bleaching; stars: hydroxyapatite clusters deposited on the surface

**Fig. 9:**
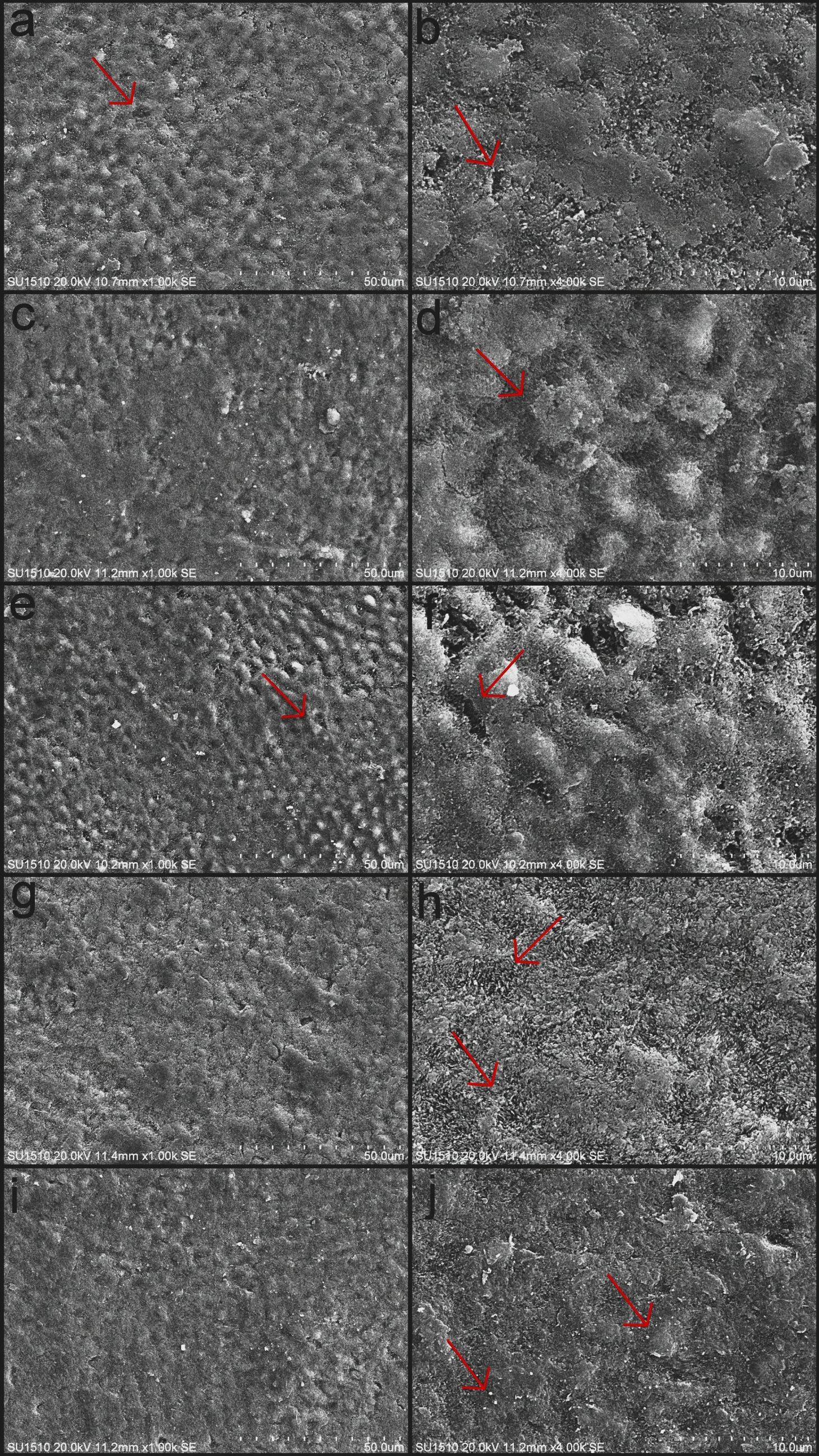
SEM micrographs of randomly selected specimens from groups G6 to G10 at × 1,000 and × 4,000 magnifications (left to right). G6 (**a**–**b**): red arrow: partial dissolution of aprismatic structure and prism cores after demineralization, irregular demineralization areas; G7 (**c**–**d**): red arrow: partial dissolution of aprismatic structure and prism cores after demineralization; G8 (**e**–**f**): red arrow: partial dissolution of aprismatic structure and prism cores after demineralization; G9 (**g**–**h**): red arrow: hydroxyapatite rods and enamel surface repair after the use of bleaching agent containing n-HAP; G10 (**i**–**j**): red arrow: hydroxyapatite clusters deposited on the surface and enamel surface repair after the use of bleaching agent containing n-HAP

Three randomly selected specimens from each study group were examined at × 1,000 and × 4,000 magnifications in the SEM. Regardless of the bleaching agent applied, the physiological structure was maintained in the sound enamel groups, with a smooth appearance of the enamel prisms and surface (Fig. 8). In the demineralization groups, an inhomogeneous and rougher structure was detected, with the dissolution of the core structure and interprismatic areas in the enamel prisms (Fig. 9). This was not clearly observed in specimens without the demineralization cycle; however, areas of n-HAP deposition on the enamel surface were distinctly visible in the demineralized groups at × 4,000 magnification.

## Discussion

This study investigated the effects in vitro of tooth-bleaching agents containing 40% HP, 40% HP + n-HAP, 16% CP, and 6% HP + n-HAP on sound and demineralized enamel tissue in terms of surface roughness and microhardness values. The changes to the enamel surface were analyzed by SEM examination. The results show that the demineralized enamel groups had considerably greater surface roughness values and significantly lower microhardness values. It was observed that tooth-bleaching agents containing n-HAP increased the microhardness values in the demineralization groups. As a result of the data obtained, the null hypothesis was rejected.

In the present study, a methodological consideration arises from comparing CP- and HP-based bleaching agents with differing concentrations. However, since CP hydrolyzes into HP (16% CP ≈ 5–6% HP), this equivalence supports the validity of comparing their effects despite compositional differences [3].

In the present study, bleaching procedures were performed in accordance with the manufacturers’ instructions to enhance clinical applicability. Although protocol standardization could minimize variability, it would compromise the intended use and real-world relevance of the products [33, 34]. Each agent is formulated with specific guidelines for optimal efficacy and safety [35]. Therefore, adherence to product-specific protocols was considered both methodologically appropriate and ethically justified to preserve external validity.

The specimens were sequentially analyzed for surface roughness, microhardness, and SEM evaluation, as designed in the present study [36]. Since a non-contact optical profilometer was used for surface roughness measurements, this procedure did not interfere with the integrity of the specimens or affect the outcomes of subsequent analyses [37, 38].

Although the Ra values are generally preferred in surface roughness studies, the Sa values also hold great importance, as they provide a more comprehensive representation of the 3D surface morphology [21, 24, 36, 39, 40]. While the Ra value represents the arithmetic mean of surface irregularities along a specific linear profile, the Sa value reflects the average height deviation calculated across the entire surface area [41–43]. Therefore, slight variations between these two values are normal and can be expected in most cases [44]. In the present study, both Ra and Sa values increased following demineralization; however, significant intergroup differences were observed only for the Ra values. This outcome may be attributed to the higher sensitivity of Ra to localized micro-irregularities, such as scratches and pits, whereas Sa, which represents an areal average, may mask such localized changes due to its broader assessment of surface heterogeneity [39, 43, 45].

Surface roughness analysis is a commonly employed quantitative method to assess enamel demineralization [23, 46]. Azrak et al. [24] reported a slight increase in surface roughness on sound enamel after applying bleaching agents with varying pH levels, whereas more pronounced effects were observed on demineralized enamel. In contrast, Cadenaro et al. [47], using neutral pH agents in an in vivo setting, found no significant change in surface roughness of sound enamel. The discrepancy was attributed to the acidic nature of the 35% CP in Azrak et al. [24]’s study. Low-pH bleaching agents are known to increase enamel roughness and reduce microhardness by softening the hard tissue [15, 21, 48]. Chow et al. [49] demonstrated that hydroxyapatite can reduce the acidity of HP solutions, which may mitigate these adverse effects. Consistently, the present study found no significant roughness increase on sound enamel with neutral or alkaline pH bleaching agents; however, surface roughness significantly increased in demineralized specimens, supporting previous findings [24].

In an in vitro study, Santos et al. [50] used a contact profilometer to evaluate the effect of Ca, F, and Ca + F added bleaching agents, followed by n-HAP paste application, on bovine enamel demineralization at days 7 and 14. While HP alone increased enamel susceptibility to demineralization, the addition of Ca or F reduced surface loss after acidic exposure. N-HAP was shown to penetrate demineralized areas, promoting remineralization by acting as a scaffold for calcium and phosphate deposition [50, 51]. However, no significant difference in enamel loss was observed between the HP-only and HP + n-HAP groups, possibly due to the high pH (8.60) of the n-HAP paste, which may limit ion release [51]. In the present study, although slight roughness increases were noted in both fluoride and n-HAP groups post-demineralization, these were not statistically significant. These findings suggest that incorporating n-HAP during, rather than after, bleaching may better support remineralization.

In the present study, upon examination of the profilometric analysis and surface topography images, the demineralized enamel surfaces treated with the home bleaching agent containing 16% CP exhibited the highest surface roughness values. This finding was supported by topographic analyses showing pronounced undulations and irregular surface patterns. The structural disintegration observed particularly in Fig. 5c suggests that prolonged application of CP may lead to significant morphological degradation of the enamel surface. Zamudio-Santiago et al. [52] similarly reported that the application of 16% CP resulted in a significant increase in surface roughness in bovine enamel, posing a potential threat to the structural integrity of the tissue. In contrast, the groups treated with bleaching agents containing n-HAP especially the 6% HP + n-HAP group exhibited less surface deterioration and better preservation of topographic integrity, which can be attributed to the surface-coating and remineralizing properties of n-HAP [8].

Enamel surface integrity was better preserved in the groups treated with n-HAP, particularly in specimens subjected to 6% HP + n-HAP. This was clearly demonstrated through non-contact optical profilometry, which allowed for precise, non-invasive surface assessment. As shown in Fig. 5e, the enamel surface exhibited a more uniform and regular topography with lower surface roughness values. These findings may reflect the synergistic action of n-HAP’s surface-coating properties and its capacity to promote remineralization. The high-resolution, non-contact nature of profilometry provided both visual and quantitative evidence of the protective effects of n-HAP on enamel structure. Consistently, Murr et al. [53] reported that n-HAP used in conjunction with low-concentration HP reduced demineralization and promoted mineral re-deposition on enamel surfaces. These findings suggest that n-HAP supplementation contributes not only to structural preservation but also to active remineralization, particularly in demineralized enamel. In sound enamel specimens, such as those shown in Fig. 4e, the application of an n-HAP-containing agent helped maintain surface continuity, with profilometric images revealing intact morphology and a homogenous distribution of possible n-HAP deposits. This observation reinforces the material’s capacity to provide a stabilizing layer on enamel. Supporting evidence from Abd et al. [54] also demonstrated that n-HAP-treated specimens demonstrated significantly reduced surface roughness and increased microhardness values, highlighting its dual protective and reinforcing function under bleaching conditions.

The initial sign of enamel demineralization is a reduction in microhardness caused by structural changes in the mineral crystals of the enamel surface [55]. For this reason, the Vickers microhardness method is frequently used to assess demineralization and remineralization in dental specimens [56–58]. Khoroushi et al. [59] conducted an in vitro study in which enamel specimens treated with 40% HP gel were subsequently exposed to three different remineralizing agents HAP, bioactive glass, and amorphous calcium phosphate. Their findings indicated that these agents, when applied in 2 g powder and 1 ml gel forms, could effectively restore microhardness. Notably, hydroxyapatite enhanced the bleaching process by promoting free radical release from HP, suggesting that incorporating n-HAP may improve both the efficacy and biocompatibility of bleaching agents.

In the present study, the bleaching agent containing n-HAP had no significant effect on the microhardness of sound enamel. This may be attributed to differences in hydroxyapatite concentration and nanoparticle size. Huang et al. [51] reported that particle size influences remineralization capacity by affecting surface area, hydration behavior, moisture retention, and solubility. Although both high and low concentrations of HP were tested in this study, no significant changes in the microhardness of sound enamel were observed. Similar findings have been reported in studies using bleaching agents with lower active ingredient concentrations [13, 19]. However, some studies have shown that high concentrations of HP can reduce enamel microhardness by promoting demineralization and degradation of organic components [60, 61]. Salinovic et al. [62] compared the effects of fluoride and n-HAP containing agents on enamel demineralized with phosphoric acid. The highest microhardness values were observed with agents containing a combination of fluoride and casein phosphopeptide–amorphous calcium phosphate (CPP–ACP). This suggests that n-HAP has potential as an alternative to fluoride containing remineralization therapies, especially given recent concerns regarding the neurotoxicity of fluoride [63]. These findings align with those of Eskelsen et al. [27] who concluded that fluoride is more effective in preventing demineralization than in promoting remineralization. Similarly, Owda and Sancakli [18] reported significantly lower microhardness values in artificially demineralized bovine enamel compared to sound enamel. However, after application of bleaching agents at different concentrations, microhardness increased slightly but not significantly except in groups treated with 40% HP. In contrast, the inclusion of n-HAP in the current study resulted in significantly higher microhardness values, confirming the remineralizing potential of n-HAP. Like other remineralizing agents, n-HAP is most effective with long-term application. The greatest increase in microhardness was observed with the use of a home bleaching agent containing n-HAP [64].

SEM examinations are preferred in demineralization studies that observe the changes resulting from bleaching and remineralizing agents such as n-HAP on the tooth surface [16, 17, 65, 66]. In the present study, SEM analysis showed that the physiological structure was preserved in the sound enamel groups, whereas an inhomogeneous and rougher structure was detected in the demineralization groups, with the dissolution of the core structure and interprismatic areas in the enamel prisms. In particular, in Fig. 8a–b (G1), the aprismatic layer on the sound enamel surface was clearly preserved, whereas in Fig. 9a–b (G6), partial dissolution of the core structure and increased surface irregularities were clearly observed following demineralization. In addition, enamel irregularities were more often seen as the peroxide percentage of the bleaching agent increased. In Fig. 9c–d (G7) and Fig. 9e–f (G8), it was observed that with increasing HP concentration, the prism structures were disrupted and the enamel surface lost its homogeneity. These findings indicate that high-concentration HP causes greater damage to the enamel structure. Similar results have been observed in studies using different concentrations of bleaching agents on sound and demineralized enamel [18, 65].

Monterubbianesi et al. [13] used SEM to examine sound tooth enamel after the application of two different agents containing 6% HP and 6% HP + n-HAP. The SEM micrograph revealed no significant change in the micromorphological structure of the enamel in the tested groups compared to the specimens without bleaching. Similarly, in the present study, Fig. 8i–j (G5) shows that although the aprismatic layer was partially lost on the sound enamel surface treated with 6% HP + n-HAP, the prismatic structure was preserved, and clusters of n-HAP adhered to the surface were marked with a star symbol. Similar to the findings of the present study, a slight increase in the irregularities in the enamel surface morphology was observed at high magnification in the 6% HP group, whereas the aprismatic layer was removed but an intact prismatic structure was preserved in the 6% HP + n-HAP group. From the perspective of the demineralized groups, the images in Fig. 9i–j (G10) demonstrated that the n-HAP containing agent promoted mineral deposition on the surface and partially restored the enamel architecture. The SEM findings clearly demonstrate both the structural protective effect of n-HAP containing agents and the morphological degradation caused by agents containing high concentrations of HP or CP on demineralized surfaces. These observations are consistent with quantitative data such as surface roughness and microhardness.

The findings of the present study suggest that home bleaching agents containing n-HAP may exert more favorable effects on the microhardness of demineralized enamel surfaces. Nevertheless, these findings do not diminish the clinical priority of enamel mineral recovery. Enamel microhardness has been shown to vary significantly depending on whether mineral recovery is achieved before bleaching procedures; therefore, completing this process prior to any bleaching intervention remains strongly recommended [54, 67]. Although bleaching agents containing n-HAP may partially attenuate the adverse effects of hydrogen peroxide and support limited surface mineral deposition during bleaching, this effect should be regarded as a short-term risk-reduction approach rather than a substitute for comprehensive or long-term therapeutic strategies [54]. From a clinical perspective, pre-bleaching interventions such as topical fluoride, CPP–ACP, or biomimetic hydroxyapatite applications enhance enamel resistance and reduce susceptibility to oxidative damage [62]. Accordingly, the use of bleaching agents containing n-HAP should be limited to situations in which pre-bleaching mineral recovery is insufficient, not feasible, or contraindicated, whereas a two-step protocol consisting of mineral reinforcement followed by controlled bleaching represents the safest and most evidence-based approach for preserving enamel structure and function during esthetic treatments. The findings of the present study should therefore be interpreted within a preventive clinical framework.

The present study has some limitations. It was conducted in vitro, and many factors that may affect demineralization and remineralization under in vivo conditions could not be included in the test protocol. Although a demineralization cycle was used to mimic clinical conditions, the effect of the agents could not be monitored in advanced demineralization or in the oral environment. Thus, further research is required to examine the effects of remineralization agents used in conjunction with or incorporated with bleaching treatments under both in vivo and in vitro conditions. Moreover, the absence of any data on color change, which is the primary objective of tooth bleaching agents, represents a significant limitation of this study. Future studies should focus on evaluating both enamel protection and bleaching efficacy to establish a clearer understanding of the balance between safety and whitening performance. Clinical in vivo studies that include color change assessment are also required to validate the laboratory findings and confirm the clinical applicability of bleaching systems containing n-HAP. Considering that n-HAP particles can influence light scattering and tooth translucency, investigating their potential impact on bleaching efficacy would be valuable in further studies.

## Conclusion

Within the limitations of this in vitro study, we offer the following conclusions:

- The tooth-bleaching procedure increased the surface roughness and reduced the microhardness of enamel exposed to demineralization.
- The effects of office and home bleaching agents containing n-HAP on sound enamel were found to be similar in terms of surface roughness and microhardness.
- The office bleaching agent containing 40% HP applied to demineralized enamel reduced microhardness.
- The home bleaching agent containing 6% HP + n-HAP applied to demineralized enamel increased microhardness.
- In the presence of initial enamel demineralization, when bleaching treatment is necessary, long-term treatments with home bleaching agents containing low concentrations of HP as well as remineralization agents such as n-HAP may be more reliable procedures than short-term treatments with agents containing high concentrations of HP. However, remineralization should ideally precede bleaching procedures to ensure enamel safety. Further clinical studies are needed to establish the most effective and safe protocols for such cases.

## Data Availability

All data supporting the findings of this study are included in the article and its supplementary materials.
